# Role of erythropoietin and its receptor in the development of endometriosis in rats

**DOI:** 10.4274/jtgga.galenos.2018.2018.0039

**Published:** 2019-02-26

**Authors:** Mehmet Yalçın Günal, Mehmet Ozansoy, Ülkan Kılıç, İlknur Keskin, Ekrem Musa Özdemir, İsmail Aslan, Zehra Eren, Cenk Ersavaş, Ertuğrul Kılıç

**Affiliations:** 1 Department of Physiology, Alanya Alaaddin Keykubat University School of Medicine, Antalya, Turkey; 2 Department of Physiology, İstanbul Medipol University School of Medicine, İstanbul, Turkey; 3 Department of Medical Biology, University of Health Sciences School of Medicine, İstanbul, Turkey; 4 Department of Histology and Embryology, İstanbul Medipol University School of Medicine, İstanbul, Turkey; 5 Experimental Animal Center, İstanbul Medipol University, İstanbul, Turkey; 6 Department of Pharmaceutical Technology, Yeditepe University School of Pharmacy, İstanbul, Turkey; 7 Department of Nephrology, Yeditepe University School of Medicine, İstanbul, Turkey; 8 Department of General Surgery, İstanbul Medipol University School of Medicine, İstanbul, Turkey; 9 Regenerative and Restorative Medical Research Center (REMER), İstanbul Medipol University, İstanbul, Turkey

**Keywords:** Endometriosis, darbepoietin, erythropoietin, MIRCERA, receptor activator

## Abstract

**Objective:**

Besides its hematopoietic function, erythropoietin (EPO) may protect tissues from degenerative disorders. As such, EPO and its receptors were revealed in nonhematopoietic cells, including stromal and endometrial epithelial cells. However, the role of EPO in endometrial disorders is still unknown. Here, we aimed to examine the role of EPO and its receptor activation in the development of endometriosis in rats.

**Material and Methods:**

Animals were treated with EPO, darbepoietin (the synthetic form of EPO) or EPO’s receptor activator, methoxy polyethylene glycol-epoetin beta (MIRCERA), after development of endometriosis. Endometriosis was induced by estrogen-administration following surgical attachment of endometrial surface on the inner abdominal wall. Treatments were started 3 weeks after induction of endometriosis and continued for the following 3 weeks. For the analysis of recurrence of endometriosis, additional analyses were conducted 3 weeks after cessation of treatments.

**Results:**

As compared with vehicle-treated animals, lesion size was reduced significantly and recurrence of endometriosis was not observed in all treatment groups. Histopathologic examination revealed that EPO and darbepoietin were more effective than MIRCERA- and vehicle-treated animals.

**Conclusion:**

Here we provide evidence that EPO is a promising candidate for the treatment of endometriosis. Our histopathologic results in particular indicate that EPO is more effective than its receptor activator MIRCERA in the development endometriosis.

## Introduction

Endometriosis is defined as an etiologically unknown gynecologic condition resulting from the displacement of cells of the endometrium toward outside the uterine cavity (1). Endometrial cells normally constitute the lining of the uterine cavity and are regulated by sex hormones. Although endometrial cells appear outside the uterus in endometriosis, they still respond to hormonal changes in a similar fashion, like the cells found inside the uterine cavity (2). Endometriosis generally occurs in reproductive years and it is found in 6-10% of women (3). The two main symptoms of endometriosis are infertility and pain; the severity of the latter depends on the menstrual cycle and it is one of the most common causes of secondary dysmenorrhea (1,4).

It is largely believed that endometriosis develops from irregularities seen in molecular cascades, where estrogen, progesterone and several prostaglandins are involved (5). One of the key pathologic features of endometriosis is inflammation, which is associated with rising levels of proinflammatory cytokines, such as interleukin (IL)-6, IL-8, and tumor necrosis factor (TNF)-α (6). Elevated levels of such cytokines would increase the adhesion of endometrial tissue onto peritoneal surfaces. Excessively synthesized estrogen and prostaglandins, and resistance against progesterone would have been used as clinically important starting points in order to develop therapeutic strategies for endometriosis (7).

Recent studies showed that erythropoietin (EPO), which is an important molecular regulator in the activation, proliferation, and differentiation of the cells of erythroid lineage, has been found to be involved in estrogen-dependent angiogenesis in the mouse uterus (8). It has also been demonstrated that EPO levels were increased in the peritoneal fluid of patients with endometriosis. As such, EPO and its receptor expressions were revealed in nonhematopoietic cells, including neurons, stromal cells, and human and mouse endometrial epithelium (9,10,11). It was revealed that EPO improves the proliferation and inhibits the apoptosis of trophoblast and decidual cells in early human pregnancy (10,11,12). In addition, it has been shown that EPO protects myocardial tissue (13), lung (14), and neuronal tissue (10,11), and participates in the recovery and remodeling of neuronal tissue after brain injury (15). As such, EPO is a promising candidate for the treatment of degenerative disorders. It is routinely administrated in patients with anemia with renal insufficiency (16); this hematopoietic factor is considered very potent and safe.

In this context, we aimed to investigate the role of EPO treatment and its receptor activation in endometriosis in rats. In addition to the use of EPO, we also used darbopoetin alpha (DARBE), which is a synthetic form of EPO, and methoxy polyethylene glycol-epoetin beta (MIRCERA), a long-acting EPO receptor activator, in order to assess their possible effects on the pathogenesis of endometriosis.

## Material and Methods

### Animal model

Forty female non-pregnant and non-ovariectomized nulligravid Sprague Dawley albino rats weighing 200-250 g were chosen for the study. They were bred for 3 to 4 months at the Experimental Research Centre. The rats were fed ad libitum and individually caged in a controlled environment (21 °C temperature and 60% humidity) with a 12-hour light/dark cycle. This study was approved by the Experimental Animals Ethics Committee (approval number: 2013/291) and the experiments were performed in compliance with the international guidelines on the ethical use of animals. The animals (10 per group) were randomly divided into 4 groups and treated with vehicle (control); EPO [for the second 3-week period, EPO (100 IU/kg) was applied intraperitoneally three times per week], DARBE (for the second 3-week period, DARBE was administered intraperitoneally once per week in 0.50 µg/kg) or MIRCERA (for the second 3-weeks period, MIRCERA was injected intraperitoneally once per week in 0.1 µg/kg).

### Experimental design and surgical procedures

All rats were anesthetized with an intramuscular administration of 60 mg/kg of ketamine (Ketalar; Eczacıbaşı İlaç Sanayi, Levent, İstanbul, Turkey) and 6 mg/kg of xylazine hydrochloride (Rompun; Bayer İlaç Sanayi, Şişli, İstanbul, Turkey).

Before starting each surgical procedure, the animals were weighed, and the weight data were statistically evaluated. 

In order to induce the endometrial foci, the uterine horns were removed from the cranial cervix, specifically, the upper bifurcation uterine region, approximately 0.5 cm caudal to the ovaries. The parametrial tissues covering the uterine horns were also removed. Each individual uterine horn was divided into two sections to get four separate tissue pieces. Each of these tissues possessing cylindrical shapes were longitudinally opened to create a linearly-shaped surface. These tissues were sutured with non-absorbable polypropylene 6-0 suture such that endometrial surfaces faced the inner abdominal wall. These four tissue pieces were implanted into a vascular area, two on the left and two on the right on the abdominal wall. Sterile saline solution was applied to the peritoneal cavity to prevent moisture loss. The midline abdominal incision was sutured using 3-0 silk sutures. The skin incision was closed in a continuous interlocking manner using 3-0 silk sutures. The sutures were supported through the use of Tensoplast adhesive bandaging (Smith & Nephew, New Zealand) for keeping the rats away from the sutured area. 

After inducing endometrial foci in all animals, estrogen (50 µg/kg) was administered twice a week for 3 weeks for the induction of endometrial foci. At the end of the first 3-week period, estrogen treatment was stopped for all groups and surgery was performed for all animals at the end of every 3-week period for 9 weeks. These surgical procedures were performed in order to assess the dimensions of the endometriotic foci in millimeters, and to randomly take one of the foci by biopsy for histopathologic analysis. The study was completed at the end of the nine weeks, and the animals were sacrificed by decapitation under anesthesia in order to evaluate recurrence of the pathology.

### Histopathologic examination

At the end of the ninth week, all animals were sacrificed in order to harvest all the endometriotic foci for histopathologic assessment. Histopathologic examination and scoring were performed by a pathologist who was blinded to the groups, and average scores were calculated when more than one lesion was observed in the same rat (17,18). 

Tissue was fixed in 10% formaldehyde and paraffin embedded. Four-micrometer-thick tissue sections were cut and stained with hematoxylin-eosin and visualized using a Nikon Eclipse light microscope. The presence of endometrial epithelium and stroma in the ectopic focus, endometrial glands, and blood vessels were used to determine the full-blown endometriosis histopathologically. Tissue sections were scored as follows: 0: Absence of endometrial epithelium; 1: Endometrial epithelial cells seen occasionally; 2: Endometrial epithelial cells well conserved; 3: Well-conserved endometrial epithelial cells and endometrial glands.

### Volumetric analysis of the endometrial foci

The dimensions of the endometrial foci were calculated according to the prolate ellipsoid formula below:

Volume (mm^3^)= 0.52 × length × width × height

### Statistical analysis

Data were evaluated using one-way ANOVA followed by LSD tests. Data are presented as mean ± standard error mean values. Throughout the study, p values <0.05 were considered significant.

## Results

In the control group, no endometriotic lesions were observed in only one rat and this rat was excluded from the study because it died in the fifth week of the study. The rest of the experimental animals remained alive until they were sacrificed. 

Body weights of all experimental animals were measured before inducing endometrial foci and every surgical procedure at the first, second and third 3-week periods. No statistically significant difference was found between and within all groups (data not shown). 

**Figure 1:**
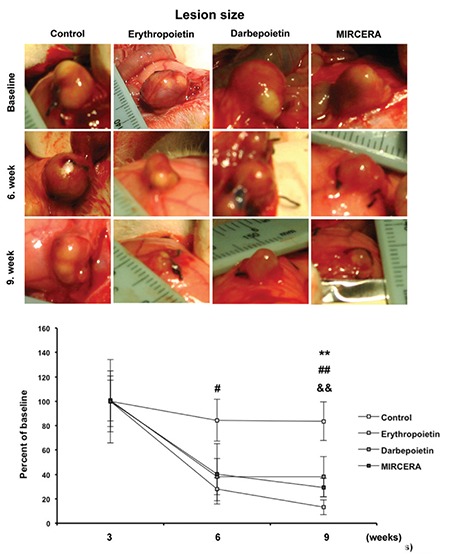
Effect of erythropoietin. Darbepoietin and MIRCERA on lesion development after induction of endometriosis. At the end of the first 3-weeks period (after stopping estrogen administration; baseline). Sizes of endometriotic foci are very similar between all groups and statistically significant differences have not been detected. In all treatment groups reduced lesion size was observed and the end of 6 weeks. No recurrence of lesions were observed 9 weeks after starting the experiment. Data are mean ± standard error mean values MIRCERA: Methoxy polyethylene glycol-epoetin beta; ^##^p<0.01/^#^p<0.05 erythropoietin compared with control; **p<0.01 darbepoietin compared with control; ^&&^p<0.01 MIRCERA compared with control

In order to evaluate the lesion sizes, the prolate ellipsoid formula was used. As expected, at the end of the first 3-week period (after stopping estrogen administration), the sizes of the endometriotic foci were very similar between all groups and no statistically significant differences were detected (Figure 1). 

On the other hand, after treating animals with the previously mentioned molecules (at the end of the second 3-week period), the lesion sizes in groups 2 (EPO), 3 (DARBE) and 4 (MIRCERA) were found to have decreased remarkably with respect to group 1 (control) (Figure 1). However, the lesion size was significantly decreased only in EPO treated animals (p<0.05). At the end of the third 3-week period (no treatment), the lesion sizes in all experimental groups were significantly lower. When a comparison of lesion sizes was made between the second and third 3-week period, the decrease in lesion sizes of group 2 (EPO) (p<0.01) and group 4 (MIRCERA) (p<0.01) seemed to be more pronounced than that of group 3 (DARBE) (p<0.01) in the third 3-week period as compared with the controls (Figure 1). 

**Figure 2:**
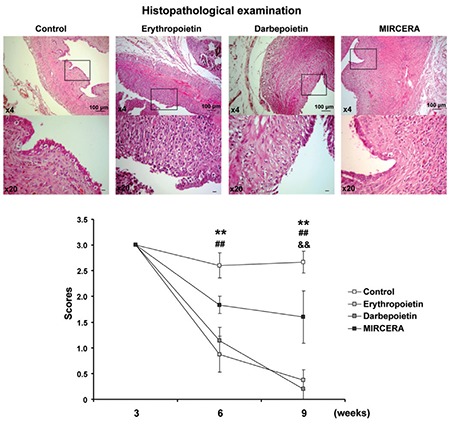
Histopathologic examination. Histopathologic examinations revealed that MIRCERA was less effective on the development of endometrial foci as compared with erythropoietin and darbepoietin MIRCERA: Methoxy polyethylene glycol-epoetin beta; ^##^p<0.01 erythropoietin compared with control; **p<0.01 darbepoietin compared with control; ^&&^p<0.01 MIRCERA compared with control

Histopathologic assessments showed that the scores of group 2 (EPO) (p<0.01) and group 3 (DARBE) (p<0.01) were significantly lower compared with the control group at the end of the second 3-week period. Scores of all experimental groups, on the other hand, were lower than the control group at the end of the third 3-week period. P values were less than 0.01 in EPO-, DARBE- and MIRCERA-treated animals as compared with the controls (Figure 2).

## Discussion

Endometriosis is a widely-occurring clinical situation seen in women with symptoms of pelvic pain and infertility. Although there are several treatment protocols for the situation, an effective cure does not exist.

EPO is an important molecule, synthesized mostly in the kidneys, liver, brain and uterus (19). In the kidneys, EPO synthesis and release is regulated by peritubular cells, whereas in the liver, hepatocytes and Kupffer cells are the main source and regulators of EPO production (20). It has been largely believed that neonatal anemia seen in early periods after birth may be caused by the reduction or complete loss of EPO synthesis and secretion from the placenta (21). It is also known that hypoxia is the primary stimulant for EPO production and its release, and hypoxia-inducible factor-1 is the main molecule in this pathway (22). 

In addition, it was shown that oxidative stress was one of the key components in the pathophysiology of endometriosis. Accordingly, scientific research for the effective treatment of endometriosis has been focused on the reduction of oxidative stress (23). In recent studies, the antioxidant activity of EPO was revealed. It was shown that EPO has the capacity to act as a biologic antioxidant, a highly potent scavenger of hydoxyl radicals, which provides a mechanistic basis encouraging proof-of-concept studies in inflammatory disorders (24,25,26). In parallel with these studies, free radical scavengers such as melatonin have been found to cause a reduction of endometriotic lesions with its antioxidant properties (24). In another study, endometriotic lesions of rats decreased in size when melatonin was applied (25). Furthermore, EPO activates PI3K/Akt signaling pathways as a leading candidate with its role in cell metabolism and survival. In contrast, Matsuzaki et al. (26) demonstrated that intraperitoneal EPO concentration increased in women with endometriosis. It was a clinical study and it is not clear whether the increased EPO level was a part of the endogenous repair process after endometriosis. 

We aimed to demonstrate the effects of exogenously-administrated EPO, DARBE, and MIRCERA on the treatment of endometriosis. We hypothesized that EPO and other agents could exert a therapeutic efficacy via their negative-feedback effect on EPO receptors, which have already been shown to play a critical role in the development of endometriosis. In this respect, we used a surgically-induced endometriosis model in rats and after induction, estrogen was administrated in order to accelerate the lesion formation, as described previously (18). In our EPO-administrated group of rats, the volumes of endometriotic foci and the histopathologic scores were both found to be decreased (p<0.05 for both). Furthermore, when the lesions were re-evaluated after 3 weeks of EPO treatment, the reduction in lesion sizes was still apparent and statistically significant. The decrease in lesion sizes and non-recurrence after stopping EPO injections led us to consider that exogenously-applied EPO could be used effectively in endometriosis.

DARBE is a structurally similar molecule to EPO but its half-life is approximately 3 times longer (~25 hours) (27). In our study, when DARBE was administrated for 3 weeks, a decrease in the sizes of endometriotic lesions was apparent but not statistically significant. However, even three weeks after stopping DARBE treatment, lesion sizes did not increase and the shrinkage of the endometriotic foci became statistically significant (p<0.05). In evaluating the histopathologic scores of the samples simultaneously, we revealed that the histopathologic scores were also lower during and after the DARBE treatment (p<0.05 for both measurements). 

In the recurrence evaluation of the endometriotic lesions three weeks after the drug-free period, the decrease of the histopathologic scores after DARBE treatment was more pronounced with respect to the decrease of scores after EPO treatment. This would be related to the longer half-life of DARBE.

The third molecule used in this study was MIRCERA, which is an activator of the EPO receptor, and its half-life is approximately 133 hours (28). When treating rats with MIRCERA, the reduction of the endometriotic lesions was macroscopically apparent but not statistically significant. The decrease in the lesion sizes continued after stopping MIRCERA treatment (period for recurrence evaluation) and this reduction was also statistically significant (p<0.05). Histopathologic assessment showed that the scores were lower than the control group but there was no statistical significance; during recurrence evaluation, on the other hand, the histopathologic scores were significantly lower than the control group (p<0.05).

It is difficult to estimate what caused the discordance between the histopathologic and macroscopic evaluations; however, if we take a closer look at the total lesion size and histopathologic scores, it is easy to demonstrate that the most effective agents were EPO and DARBE. Not surprisingly, we have shown that DARBE was therapeutically superior to EPO in decreasing the recurrence rate. This is likely due to the relatively long-lasting effect of DARBE in comparison with EPO. 

As compared with EPO, MIRCERA exerts different activity at the receptor level, which is defined as a slow association and rapid dissociation from the EPO receptor. Although there are strong data suggesting the antioxidant and anti-inflammatory activity of DARBE and EPO, there are still insufficient preclinical and clinical data to indicating the strong antioxidant effect of MIRCERA (29,30,31). To the best of our knowledge, there have been very few studies in other disciplines that have suggested the free radical scavenger effect of MIRCERA. Taken together, it is not unreasonable to assume that the therapeutic failure of MIRCERA could be partly attributed to its low anti-oxidant activity which needs to be confirmed with further comparative studies on the antioxidant effect of EPO, DARBE and MIRCERA. 

In the light of these observations, endometriotic lesions in rats were found to be decreased in size when EPO, DARBE, and MIRCERA were administrated. Moreover, these molecules seemed to reduce the recurrence rate of lesions after the treatment period. It is probable that the mechanism of action of these molecules could be related to the antioxidant properties of EPO itself and/or molecular pathways in which EPO might play its role. It should also be emphasized that large-scale clinical investigations on women with endometriosis are needed to be implemented before these three molecules can begin use in the treatment of endometriosis.
